# Families of Nuclear Receptors in Vertebrate Models: Characteristic and Comparative Toxicological Perspective

**DOI:** 10.1038/srep08554

**Published:** 2015-02-25

**Authors:** Yanbin Zhao, Kun Zhang, John P. Giesy, Jianying Hu

**Affiliations:** 1 MOE Laboratory for Earth Surface Processes, College of Urban and Environmental Sciences, Peking University, Beijing 100871, China; 2 Department of Veterinary Biomedical Sciences and Toxicology Centre, University of Saskatchewan, Saskatoon, Saskatchewan, Canada; 3 Department of Zoology, and Center for Integrative Toxicology, Michigan State University, East Lansing, MI, USA; 4 Department of Biology & Chemistry and State Key Laboratory in Marine Pollution, City University of Hong Kong, Kowloon, Hong Kong, SAR, China

## Abstract

Various synthetic chemicals are ligands for nuclear receptors (NRs) and can cause adverse effects in vertebrates mediated by NRs. While several model vertebrates, such as mouse, chicken, western clawed frog and zebrafish, are widely used in toxicity testing, few NRs have been well described for most of these classes. In this report, NRs in genomes of 12 vertebrates are characterized via bioinformatics approaches. Although numbers of NRs varied among species, with 40–42 genes in birds to 66–74 genes in teleost fishes, all NRs had clear homologs in human and could be categorized into seven subfamilies defined as NR0B-NR6A. Phylogenetic analysis revealed conservative evolutionary relationships for most NRs, which were consistent with traditional morphology-based systematics, except for some exceptions in Dolphin (*Tursiops truncatus*). Evolution of PXR and CAR exhibited unexpected multiple patterns and the existence of CAR possibly being traced back to ancient lobe-finned fishes and tetrapods (Sarcopterygii). Compared to the more conservative DBD of NRs, sequences of LBD were less conserved: Sequences of THRs, RARs and RXRs were ≥90% similar to those of the human, ERs, AR, GR, ERRs and PPARs were more variable with similarities of 60%–100% and PXR, CAR, DAX1 and SHP were least conserved among species.

Nuclear receptors (NRs) are one of the largest groups of transcription factors in vertebrates, and serve important functions in regulation of a range of physiological functions including growth and differentiation of cells, metabolic processes, reproduction, development and overall homeostasis. Transcriptional activities of NRs are regulated by binding of endogenous small lipophilic compounds^1^,^2^. There is growing evidence that diverse chemicals that occur in the environment, including synthetic molecules such as pharmaceuticals, endocrine disrupting chemicals and some industrial compounds, can mimic endogenous small compounds that can bind to ligand binding domains (LBDs), activate NR-mediated signals that then lead to toxic responses^3^,^4^. Typically, interactions of some pesticides and industrial chemicals with estrogen (ER) and androgen (AR) receptors have been linked to a number of adverse effects including birth defects, developmental neurotoxicity, both male- and female-factor reproductive health, such as decreased quality of sperm, and increased incidences of cancers^5^,^6^,^7^.

A series of *in vitro* bioassays, based on signaling of endocrine receptors including well-studied steroid hormone receptors such as ER, AR, glucocorticoid receptors (GRs), and progesterone receptor (PR) and the less well-studied retinoic acid receptor (RAR), retinoid X receptor (RXR), and thyroid hormone receptor (THR), have been established or are under assessment by OECD and/or US EPA^8^,^9^,^10^. Due to their relatively clear physiological functions and responses to environmentally-relevant organic micropollutants, these NR-based assays have been used in assessment of toxicological effects of chemicals in the environment. For example, ERs, AR and THRs, involved in development and maintenance of the endocrine system, have been demonstrated to be targets of alkylphenols, phthalates (PAEs), dichlorodiphenyltrichloroethane and some metabolites of polychlorinated biphenyls (PCBs) and polybrominated diphenyl ethers (PBDE)^11^,^12^,^13^. Besides endocrine receptors, PXR and CAR, NRs that participate in metabolism of both endobiotics and xenobiotics to detoxify or bioactivate chemicals, can be activated by a variety of pharmaceuticals such as rifampicin, pesticides such as chlorpyrifos and methoxychlor, and other synthetic chemicals used in industry, such as PBDEs and BPA^14^,^15^,^16^,^17^ In addition to these well-known NRs, there are more NRs, that, during the past decade, have been identified in genomes of several vertebrates. These include 48 NR genes in human (*Homo sapiens*), 47 genes in rat (*Rattus norvegicus*), 49 genes in mouse (*Mus musculus*) and 68 genes in the teleost puffer fish *Fugu rubripes*^18^,^19^. Specifically, structures of 48 NRs in the human have been identified and categorized, based on sequence homology, into seven different subfamilies NR0B-NR6A^20^. Except for two NRs in the subfamily NR0B which lack a DNA binding domain (DBD), all 46 NRs contain the following six functional domains: (A–B) variable N-terminal regulatory domain; (C) conserved DNA-binding domain; (D) variable hinge region; (E) conserved ligand binding domain (LBD) and (F) variable C-terminal domain^20^. In addition, sets of NRs described in humans offered a better understanding of characteristics of NRs, and provided insight for uncovering novel molecular and signal targets and mechanisms of action of synthetic toxicants. For instance, it has been found that some widely used pharmaceutical drugs that are found in the environment, including thiazolidine diones, trichloroacetic acid and toxaphene are ligands for human RORα, PPARα and ERRα, respectively^21^,^22^,^23^. Compared with the extensive understanding of NRs in human, fewer NRs have been identified in other vertebrates used as models to screen chemicals for toxic potencies, such as reptiles, amphibians and teleost fishes. While in recent years, due to extensive information about their developmental biology and molecular genetics and now the availability of completed sequencing of their genomes, these vertebrate species have been much used as toxicological models such as western clawed frog (*X. tropicalis*), zebrafish (*Danio rerio*), and freshwater Japanese medaka (*Oryzias latipes*)^24^,^25^,^26^, information on NRs in these vertebrates were still limited to ERs, AR, GR, PXR, RARs and PPARs, though studies on some novel NRs, such as VDR, FXR and NURR are in progress^27^,^28^,^29^. Additionally, since sets of NRs in human, mouse and rat that have been identified in previous studies were based on their genomes assembled a decade ago^18^, there is also a need to reevaluate the characteristics of NRs in these genomes due to the constantly updated sequence data and annotations. In addition to the sequences of genomes, predicted transcriptomes and proteomes, now available for all of these species in Genebank and Ensembl, provide useful databases that can be further used to uncover and characterize additional NRs. Therefore, comprehensive descriptions of NRs and their families for these vertebrates used as models to screen for toxic potencies of chemicals, will be helpful for their further development and interpretation of results of studies of synthetic chemicals of environmental significance.

In this study, complete sets of NRs were described for genomes of 12 vertebrates used as models in studies of toxic potency and mechanisms of action of chemicals. Several bioinformatics approaches were applied to four mammals (human, *Homo sapiens*; mouse, *Mus musculus*; rat, *Rattus norvegicus* and dolphin, *Tursiops truncatus*), two birds (chicken, *Gallus gallus* and mallard (wild duck), *Anas platyrhynchos*), a reptile (Chinese softshell turtle, *Pelodiscus sinensis*), an amphibian (Western clawed frog, *Xenopus tropicalis*) and four teleost fishes (zebrafish, *Danio rerio*; medaka, *Oryzias latipes*; tilapia, *Oreochromis niloticus* and stickleback, *Gasterosteus aculeatus*). The locations of NRs on chromosomes, phylogenetic analysis and DBD and LBD sequence conservations among species were also analyzed to better understand the characteristics of these NRs in these vertebrates.

## Results and Discussion

### Identification of NRs in 12 vertebrates

**Figure 1:**
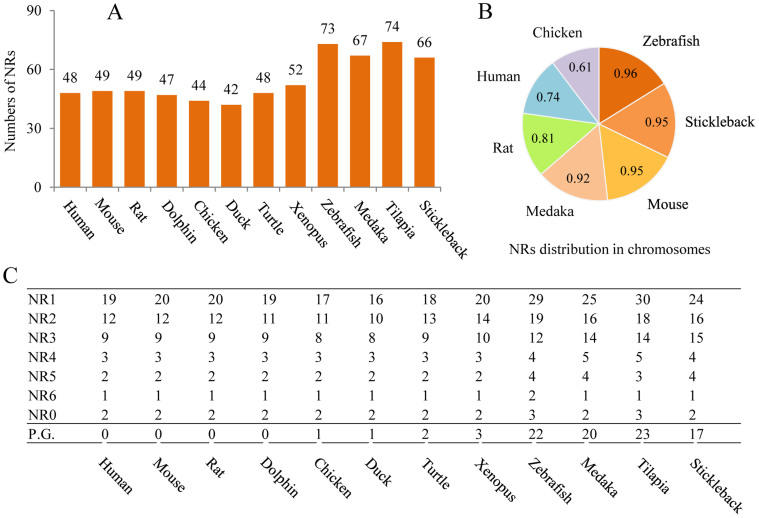
Identification of NRs in genomes of 12 toxicological vertebrate models. (A) Total number of NRs in each vertebrate genome (B) the genomic distributions of NRs in seven vertebrate species (C) the number of NRs for each type (NR0B-NR6A) and the paralogous gene numbers (P.G.) in total.

**Figure 2:**
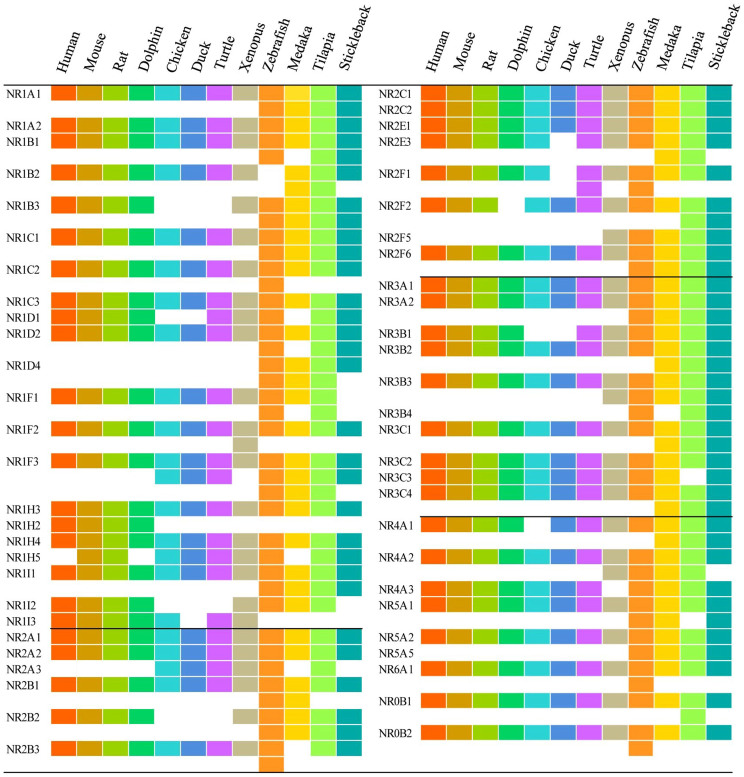
Nuclear receptor families in 12 model vertebrates. Each nuclear receptor is presented as a colored block. The white spaces indicate that no ortholog was identified. Nuclear receptor family for each vertebrate species was marked with different color. From left to right: human “
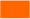
”; mouse “
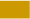
”; rat “
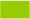
”; dolphin “
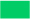
”; chicken “
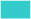
”; duck “
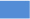
”; turtle “
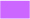
”; frog “
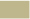
”; zebrafish “
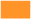
”; medaka “
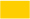
”; tilapia “
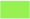
” and stickleback “
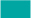
”.

Substantial and continuous information gathered from developmental biology and molecular genetics, together with the complete sequencing of genomes has placed a series of vertebrate species in attractive positions for use in toxicological research. Twelve species were chosen for description and complete sets of NR genes within their genomes were identified by use of a systemic bioinformatics approach. In total, 42–74 NR genes were uncovered within these vertebrates and a large number of variations were observed among classes (Fig. 1A, Table S2). Comparisons of sequences showed that all of these NRs displayed significant similarity to NRs of the human and could be categorized into the seven subfamilies NR0B-NR6A, with no novel subfamilies. For mammals, there were 48, 49, 49 and 47 NRs identified in human, mouse, rat and dolphin genomes, respectively (Fig. 1A). Compared to the human, one more gene (NR1H5) was observed for mouse and rat and one (NR2F2) was absent from dolphin (Fig. 2). Sets of NRs in human and mouse were consistent with previous reports^18^, while two more NRs (NR1D2 and NR2E3) were newly identified for the rat. The absences of these two NRs in rat in previous study^18^ were due to the existence of sequence gaps in the rat genome which was assembled in 2003.

**Figure 3:**
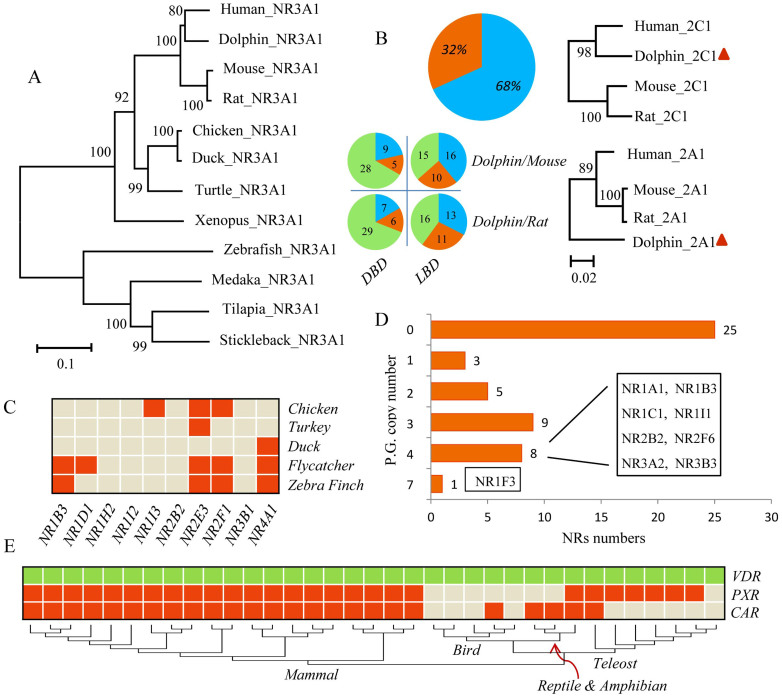
Characteristics of the 12 NRs families. (A) Phylogenetic tree for 12 NR3A1 (ERα) genes (B) The evolutionary relationships of NRs among dolphin, rodents and human species. Left: the proportions of dolphin NRs with closer relationships with human compared to rodents are presented as percent/number and blue colour. The proportions of rodents NRs with closer relationships with human are presented as percent/number and orange colour. Green colour represents the NRs numbers with equivalent sequence similarities with human for dolphin and rodents. Right: phylogenetic tree for NR2C1 and NR2A1 represents the different positions of NRs for dolphin. (C) Comparative searches for the ten lacked NRs in five bird species (D) Paralogous gene copy numbers for each type of NRs (E) Comparative searches for NR1I genes (VDR, PXR and CAR) in 35 vertebrates, including 20 mammals, 5 birds, 2 reptiles, 1 amphibian and 7 teleost (details are described in Table S4). Phylogenetic tree was developed utilizing 35 full amino acid sequences of VDR.

The numbers of NRs in birds were less than those in human, though there were some unique genes observed. There were seven NRs (NR1B3, NR1D1, NR1H2, NR1I2, NR2B2, NR3B1 and NR4A1) present in the human that were absent from the chicken. Similarly, there were nine NRs (NR1B3, NR1D1, NR1H2, NR1I2, NR1I3, NR2B2, NR2E3, NR2F1 and NR3B1) present in the human that were absent from the mallard, though there were three new NRs (NR1F3, NR1H5 and NR2A3) were identified that were unique to chicken and mallard (Fig. 2). Similar absences were observed in the genomes of turkey (*Meleagris gallopavo*), flycatcher (*Ficedula albicollis*) and zebra finch (*Taeniopygia guttata*), where 9, 5 and 6 NRs, respectively, that are present in the human genome were absent from these birds (Fig. 3C). These results demonstrated that a cluster of NRs were indeed absent from genomes of the class aves, especially in galloanserae, that were deleted during the course of evolution.

Some NRs present in the human were absent from turtle and western clawed frog while some others were unique in these species. In the one species of turtle, 48 NRs were identified with four genes absent (NR1B3, NR1H2, NR1I2 and NR2B2) and four new genes gained (NR1F3, NR1H5, NR2A3 and NR2F1) compared with those in human. Similarly, 52 NRs were identified in western clawed frog with 2 genes absent (NR1H2 and NR4A3) and six additional genes (NR1F2, NR1H5, NR2A3, NR2F5, NR3B3 and NR4A2) appeared which were not present in the human (Fig. 2).

For the four teleost fishes studied, there were many additional NRs uncovered in this study. Specifically, 73 and 74 NRs were identified in zebrafish and tilapia, respectively (Fig. 1A), which were consistent with those reported for *Fugu rubripes* (68 NRs identified)^19^. The additional NRs were mainly due to the paralogue genes exist in their sets of NRs (Fig. 1C). In zebrafish, two or more paralogues were identified to correspond with one of 20 NRs in human and with one of 18, 22 and 17 NRs in medaka, tilapia and stickleback, respectively. Existences of paralogue genes in teleost fishes were not random but focused on some specific NR units. For instance, NR1F3 (RORγ) was the most abundant NR, with a total of seven paralogue gene copies in these four teleost fishes. The NRs NR1A1, NR1B3, NR1C1, NR1I1, NR2B2, NR2F6, NR3A2, and NR3B3 were also rich in paralogues, with one paralogue gene copy in each of the four teleosts (Fig. 3D).

### Characteristics of NRs families

Genomic locations of NRs in seven vertebrate genomes (human, mouse, rat, chicken, zebrafish, medaka and stickleback) were retrieved via the Ensemble annotations. In general, distributions of NRs on chromosomes were more widespread in teleost fishes than those of mammals and birds (Fig. 1B). This is possibly due to the existence of more paralogue genes in teleosts. For example, NRs in zebrafish, medaka and stickleback were distributed throughout their genomes except for 1–2 chromosomes. The most abundant clusters of NRs were observed on chromosomes 8 and 16 in zebrafish, each with 6 NRs; on chromosomes 7 and 16 in medaka, each with 7 NRs; and on chromosome 12 in stickleback, with 8 NRs. The narrowest distribution of NRs was observed for species of chicken, in which 44 NRs were distributed in 61% (19/31) chromosomes.

**Figure 4:**
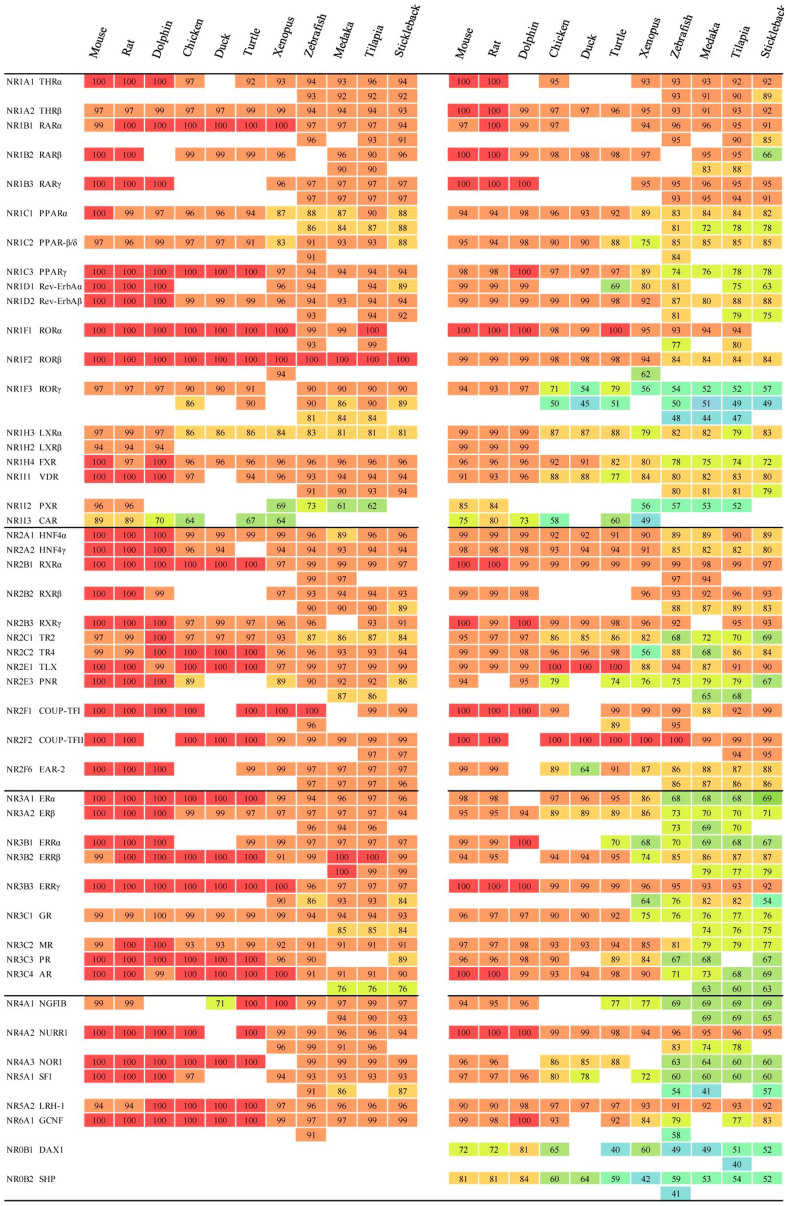
Pairwise alignments between DBD/LBD amino acid sequences of 48 human NRs and the corresponding orthologs in other eleven vertebrate species. Left for the DBD sequence comparisons and right for the LBD. The sequence similarities are presented as the percentage (%) and relevant color. NRs, with incomplete amino acid sequences of DBD/LBD, were not included in this comparison.

**Figure 5:**
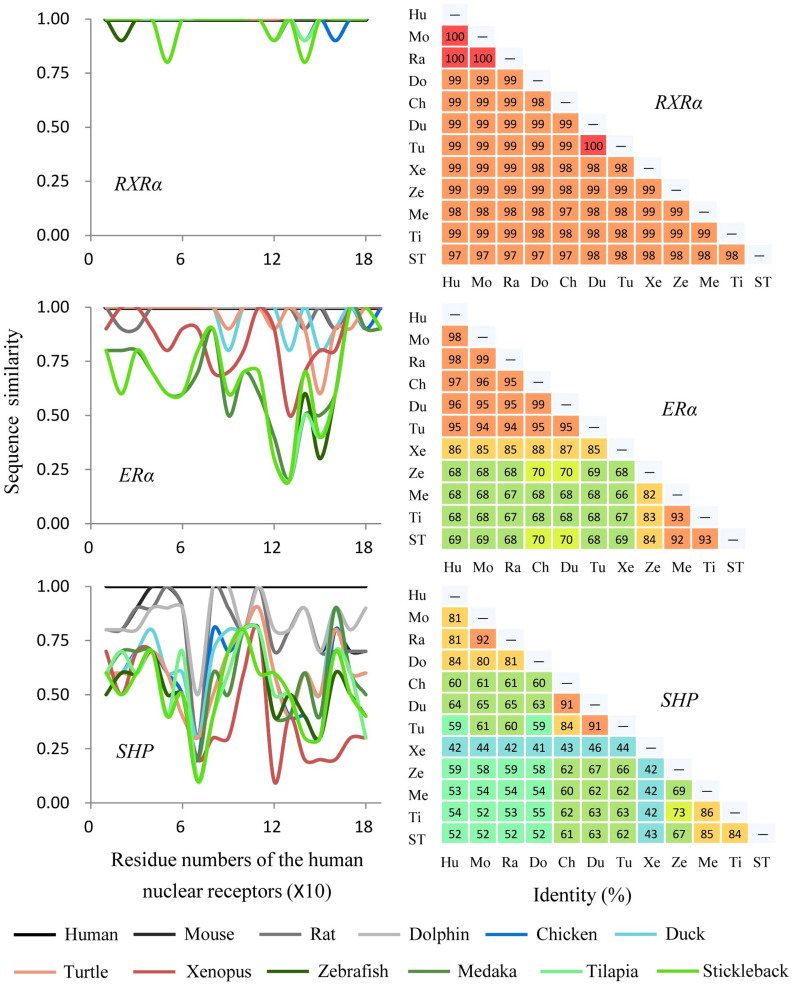
Variations in LBD sequence conservation across the sequence of RXRα, ERα and SHP. Left: LBD sequences for eleven vertebrates compared to the related human nuclear receptors. All sequences were window averaged across 10 residues. Right: multiple sequence alignments among the 12 vertebrates. The sequence similarities are presented as the percentage (%) and relevant color. The LBD sequence of ERα in Dolphin was not included in this comparison due to the incomplete amino acid sequences.

Phylogenetic analyses, based on their full amino acid sequences and DBD plus LBD compositions of NRs, were performed for 48 types of NRs among these 12 vertebrates. The Neighbor-Joining (NJ) and Maximum-Likelihood (ML) phylogenetic analyses showed similar patterns, while the Neighbor-Joining algorithm gave better resolution at the base of the phylogram. Conservative evolutionary relationships were observed for most NRs, i.e. the evolutionary relationships were generally consistent with the traditional morphology-based systematics (Fig. S1). As exemplified for NR3A1 (ERα), closer relationships were observed within each class and the traditional teleost-amphibian-reptile-bird and mammal evolutionary relationships were followed (Fig. 3A). This was verified by the similarity of sequences of the LBD of ERα among species (Fig. 4). In details, about 82–93% sequence similarities among teleost, 99% between birds and 98–99% among mammals was observed and the sequence similarities among classes were relatively small (Fig. 5). Some exceptions were observed in Dolphin such as NR2A1 and NR2A2 (Fig. S1). Though dolphin, diverged from artiodactyls approximately 50 million years ago^30^, was thought to show the closest relationship with human among the 12 vertebrates, there were 32% NRs that showed closer relationships between rodents and human compared with those in dolphin. Similarities between sequences of the DBD and LBD also confirmed this likely historical divergence. In rodents, 13% of sequences of amino acids of DBD and 26% of those of the LBD exhibited relationships more similar to those of the human than dolphin (Fig. 3B). These variations in NRs in dolphin were possibly due to the results of positive Darwinian selection, the major driving force for adaptive evolution and diversification among species, to adapt their radical habitat transition from land to a marine environment. Though increasing toxicological research has been preformed using dolphins and extrapolations from dolphin to human were thought to be more significant, results of the present study demonstrated more variations, indicating more genetic characteristics should be taken into account when assessing toxicities of chemicals based on results of studies with dolphins. In addition, since PXR and CAR displayed the largest variations and were absent in several vertebrates used in this study (Fig. 2 and 4), more comparisons among species were conducted. Existence of NR1I (VDR, PXR and CAR) genes were demonstrated in 35 vertebrate species (20 mammals, 5 birds, 2 reptile, 1 amphibian and 7 teleost fishes) with for which complete sequences of genomes were available and unexpected patterns were showed for their evolutions. VDR genes appeared in all vertebrate genomes, a result which was consistent with those in previous reports that VDR could be detected in mammals, birds, amphibians, reptiles, teleost fishes, and even the sea lamprey^31^. PXR appeared in most teleost fishes (expect for stickleback), amphibians and mammals (also known as SXR), but were totally absent from reptiles and birds. Though CAR also appeared in all mammals, it exhibited quite different patterns in other classes. CAR was mostly absent in birds (expect for chicken), but retained in reptiles and amphibians, and appeared in lobe-finned fishes and tetrapods (Sarcopterygii) (Fig. 3E). Since Sarcopterygii appeared nearly 400 million years ago during the Devonian, and are widely accepted as ancestors of all tetrapoda, including amphibians, reptiles, birds and mammals^32^, the appearance of CAR in Sarcopterygii possibly indicated that the existence of CAR was much earlier than previously thought. In general, these results revealed a novel evolutionary relationship for PXR/CAR. These two NRs likely coexisted in ancient Sarcopterygii, first due to the duplication events, descended into amphibians and then to mammals, but one of them was absent from reptiles and both were absent from most birds (Fig. S2).

### Alignment of sequences of DBD and LBD

Since cross-species extrapolations from surrogate vertebrate species to humans are usually considered to be crucial for human risk assessment of chemicals, better understanding of similarities of these NRs sequences among species will be useful to facilitate these extrapolations and better understand the toxicities of environmental chemicals. In the present study, pairwise alignments were constructed between sequences of DBD/LBD of 48 human NRs and their corresponding orthologs in the other eleven vertebrate species (Fig. 4). As expected, DBDs of the orthologous proteins generally shared relatively great conservation with sequences in human (Fig. 4, left), especially, for the mouse, rat and dolphin, in which 94%–100% sequence similarities were observed for most NRs, expect CAR (70%–89%), and almost 70% (31/46, 32/46 and 31/42, respectively) orthologous proteins showed 100% similarities with sequences of the human. For bird, reptile, amphibian and teleost fishes, most NRs also displayed conservation of sequences (usually >90%), especially for RORβ (100% for all species). While there are also some exceptions, such as PXR (61%–73%), CAR (64%–67%), and PPARα and TR2 in teleost (87%–90% and 84%–87%, respectively), which indicates potential alternations on target genes and signals for these NRs among vertebrate species.

Compared to the more conserved sequences of DBD regions of NRs among species, sequences of the LBD displayed more variation. The greatest variation was observed for DAX1 (40%–81%), while the least variation was observed for COUP-TFII (99%–100%) compared with those in human (Fig. 4, right). To our best knowledge, this is the first time all NRs LBD have been compared among vertebrates, which showed a broader and novel insight to investigate the LBD differences between species and between multiple NRs units. In the present study, three groups were identified in general based on similarities in sequences of NRs. The first group contained 13 NRs including THRα, THRβ, RARα, RARβ, RARγ, RORα, RXRα, RXRβ, RXRγ, COUP-TFII, ERRγ, NURR1 and LRH1 (except some orthologs for RARα, RORα, RXRβ, RXRγ and NURR1) with ≥90% similarity of sequences of the LBDs for all eleven vertebrates compared with those of the human (Fig. 4, right). As observed for RXRα, 97–100% similarities in sequences, for the best alignment orthologs, were observed from multiple sequence alignment (Fig. 5). Variations in conservation of sequences, window averaged across 10 amino acid residues, found that there were fewer than 5 variations in amino acid residues among these 12 vertebrate species, and most of them were observed in α-helix 3 to α-helix 6 of the LBD structures (Fig. 5). RXRα commonly functions as a heterodimers with other NRs and mainly mediates signaling of hormones derived from vitamin A (retinol) such as 9-cis retinoic acid, and are involved in multiple physiological functions of vertebrates such as embryonic patterning and organogenesis, proliferation of cells and differentiation of tissues^33^. It has been reported that among vertebrates, such as mouse and human, LBDs of RXRα interacted with similar types of ligands with similar binding affinities^34^,^35^. Sequence similarities of these 13 NRs among vertebrates suggested potential straightforward interspecies extrapolations when assessing toxicity of chemicals via these NRs. Approximately 77% of NRs such as the well-known ERs, AR, PR, PPARs and VDR can be sorted into the second group, exhibiting 60–100% similarities of sequences (for the best aligned orthologs) compared with those of human. Similarities in sequences of these NRs among four fishes were substantially the same and usually ≥90% in mouse, rat and dolphin, showing apparent differences in sequences of amino acids between teleosts and mammals. Specifically, LBDs of NRs in the second group, such as ERα and PPARγ, always shared the same variations in amino acids within four fishes, which were quite different from those of mammals (Fig. 5 for ERα). ERα is a well-studied NR, activated by endogenous and exogenous estrogens, and plays a variety of central physiological roles, such as maintenance of reproductive, cardiovascular and central nervous systems in vertebrates^36^. Potencies of binding of ligands to LBDs of ERα were different for fishes when compared to mammals. It has been reported that widespread chemicals like 4-t-octylphenol and bisphenol A (BPA) bound with greater avidity to rainbow trout ER than that of human or rat. Also, types of ligands were various: of 34 chemicals tested, 29 can bind to ER of rainbow trout, while only 20 of them can bind to ER of human/rat^37^. PPARγ is also a well-studied transcription factor, which could be activated by fatty acids and is involved in lipid and glucose metabolism^38^. Reports on binding strengths of LBDs for PPARγ were rare, but interspecies extrapolations on LBD binding activities can be likely to estimate, due to the similar sequence characteristics between PPARγ and ERα. In the third group, with less than 85% similarities in sequences of eleven vertebrate species compared with those in human, four NRs including PXR, CAR, DAX1 and SHP (Fig. 4) were classified as being different from human. DAX1 and SHP, which belong to the subfamily NR0B, displayed the greatest variations among NRs and among vertebrates (Fig. 4 and 5), a result which is consistent with those reported previously that NRs in the NR0B group were a unique class of NRs with among-species variability in sequences and lacking DBD domains^18^. PXR and CAR were also assigned to this group, and exhibited apparent differences among vertebrates and even among fishes. PXR and CAR can be activated by xenobiotics and have relatively broad abilities to bind ligands^39^. The unusually great diversity in sequences of the LBD among species could be related to diversity in binding activities among species. This is exemplified by the fact that phenobarbital, a pharmaceutical that is generally detectable in effluents of municipal waste water plants (WWTP), was a moderate activator of the zebrafish PXR and exhibited greater binding affinity with human PXR, while it did not bind to PXR of mouse^39^. These differences among species might be due to the differences in diet and physiology among vertebrates, and such largely differences of sequences of PXR and CAR among vertebrates complicated the *in silico* extrapolations.

Here, for the first time, genes that code for NRs and their relative characteristics are provided for 12 vertebrate species used as model animals in screening of toxic potencies of chemicals. These results will help understanding of the NRs in vertebrates and will be useful for clarifying mechanisms of toxic effects of environmental chemicals on these model species and also the extrapolations from the effects on these surrogates to human.

## Methods

### Identification of NRs in 12 vertebrate genomics

Identification of sequences for NRs was performed as described previously^40^,^41^ with slight modifications. In brief, the putative NRs for each vertebrate were identified through a combination of BLASTn and BLASTp searches of the genome and protein databases, which were obtained from NCBI and Ensembl. The nucleotide and protein sequences of 165 described NRs in three vertebrates (48 in human, 49 in mouse and 68 in *Fugu rubripes*) were downloaded from GenBank and used as templates for interrogating the vertebrate databases. Nucleotide homology searches were performed using the full nucleotide sequences of each of the 165 NRs against these 12 genomic sequences database at NCBI by use of nucleotide BLAST with a blastn algorithm and an e value cut off of 1e-04. Protein sequences were then used to construct multiple sequence alignments by ClustalX2 (http://www.clustal.org/clustal2/) and then the DNA-binding domain (DBD) and the ligand-binding domain (LBD) amino acid sequences were demonstrated. BLASTp searches were performed using the conserved DBD plus LBD domains against the non-redundant vertebrate protein sequence database at NCBI by use of protein BLAST with a blastp algorithm and an e value cut off of 1e-25. The e cut-off values were set to be just loose enough to find all the *Fugu* NRs when using human NRs as queries. Genes identified by BLASTn and BLASTp searches were then combined and individual putative genes were sorted according their unique DNA and amino acid sequences. All these putative genes were verified by online software NRpred and iNR-PhysChem to remove the false-positive hits, and the NR0B1 and NR0B2, which are known to lack the DBD region, were added to the final sets of NRs. Details for the sequence searches were shown in Table S1. Finally, complete sequences for each NR in each vertebrate species were loaded into Ensembl database. The nomenclatures of NRs were based on Ensembl's GeneTree and Orthology annotations.

### Genomic distributions

Genomic location for each nuclear receptor in seven vertebrate genomes (human, mouse, rat, chicken, zebrafish, medaka and stickleback) were retrieved via the Ensembl annotations, and then mapped onto complete vertebrate karyograms.

### Analyses of sequences of DBD and LBD

Sequences of peptides in the DBD and LBD domains for each NR were identified by use of Pfam software (http://pfam.sanger.ac.uk/, Pfam 27.0) and modified manually, based on characteristics of DBD and LBD regions reported previously. The sequence of DBD, which is classified as a type-II zinc finger motif, corresponds to a 75–80 amino acid residue segment, starting at the location of two amino acid residues before the first conserved cysteine and encompassing both C4 zinc fingers and the LBD, a flexible unit made of α-helices containing of 170 to 210 amino acid residues, begin at the 12th residue of α-helix 3 and extended through α-helix 10^42^,^43^.

The pairwise alignments between sequences of the DBD and LBD of human protein and corresponding orthologs in the other 11 vertebrates were constructed by use of the NCBI BLASTp software with default parameters. Similarities in sequences were calculated based on the numbers of identical residues over the total numbers of aligned residues in human.

### Phylogenetic analysis

Phylogenetic trees were constructed by use of amino acid sequences of 48 types of NRs downloaded from Ensembl based on the set of homologous NRs in the human. Only full- length molecules were included for the analysis. Some genes without complete amino acid sequences in the Ensembl database were retrieved from NCBI/EMBL/DDBJ databases (Table S3). They were also included. The Ensembl ID of each NR used in the analyses is available in SI Table S2. Conserved sequences of DBD and LBD for each NR were also isolated and used as a supportive analysis. Sequences of DBD and LBD were combined and then aligned, except for NR0B1 and NR0B2. Multiple alignments of sequences of amino acids were generated by use of ClustalX2 software with default parameters, and the results used for construction of phylogenetic trees by implementation of the Neighbour-Joining and Maximum-Likelihood algorithms with a Poisson model in MEGA6 software (http://www.megasoftware.net/mega.php). Confidence for branching patterns was assessed by bootstrap analysis (1000 replicates). For NR1I1 (VDR) analysis, the full amino acid sequences of NR1I1 in 35 vertebrates, including 20 mammals, 5 birds, 2 reptiles, 1 amphibian and 7 teleost fishes (Table S4), were downloaded from the Emsenbl database. These full amino acid sequences were then aligned and applied for gene phylogenetic analysis by use of the same method described above.

## Author Contributions

Y.B.Z. and J.Y.H. designed the experiments, Y.B.Z. and K.Z. performed the experiment and analyzed the data, Y.B.Z., K.Z., J.P.G. and J.Y.H. wrote the manuscript. All authors contributed to scientific discussions of the manuscript.

## Supplementary Material

Supplementary Information Supplementary information
