# Supplementary material for: Families of Nuclear Receptors in Vertebrate Models: Characteristic and Comparative Toxicological Perspective
Source: Sci Rep. 2015 Feb 25;5:8554. doi: 10.1038/srep08554 (PMC4339804; doi:10.1038/srep08554)
Supplement: Supplementary Information [file srep08554-s1.pdf]

Supplementary information for:

Families of Nuclear Receptors in Vertebrate Models: Characteristic and Comparative Toxicological Perspective

Yanbin Zhao[1], Kun Zhang[1], John P. Giesy[2,3,4], and Jianying Hu[1]

[1]MOE Laboratory for Earth Surface Processes, College of Urban and Environmental Sciences, Peking University, Beijing 100871, China

[2]Department of Veterinary Biomedical Sciences and Toxicology Centre, University of Saskatchewan, Saskatoon, Saskatchewan, Canada

[3]Department of Zoology, and Center for Integrative Toxicology, Michigan State University, East Lansing, MI, USA

[4]Department of Biology & Chemistry and State Key Laboratory in Marine Pollution, City University of Hong Kong, Kowloon, Hong Kong, SAR, China

Address for Correspondence

Dr. Yanbin Zhao; Prof. Dr. Jianying Hu

College of Urban and Environmental Sciences

Peking University, Yi Fu Second Building

Beijing 100871 China

TEL & FAX: 86-10-62765520

Email: zhaoyb@pku.edu.cn; hujy@urban.pku.edu.cn

Figure S1. Phylogenetic analysis for 48 types of nuclear receptor genes in twelve vertebrate species. Numbers at branches indicate the bootstrap probabilities (≥90%) with 1,000 replicates. Neighbour-Joining trees of ClustalX-aligned full amino acid/DBD plus LBD sequences were constructed and displayed for the majority of NRs. For some trees, which displayed better topological structures in Maximum-Likelihood analysis, the ML trees were constructed instead.

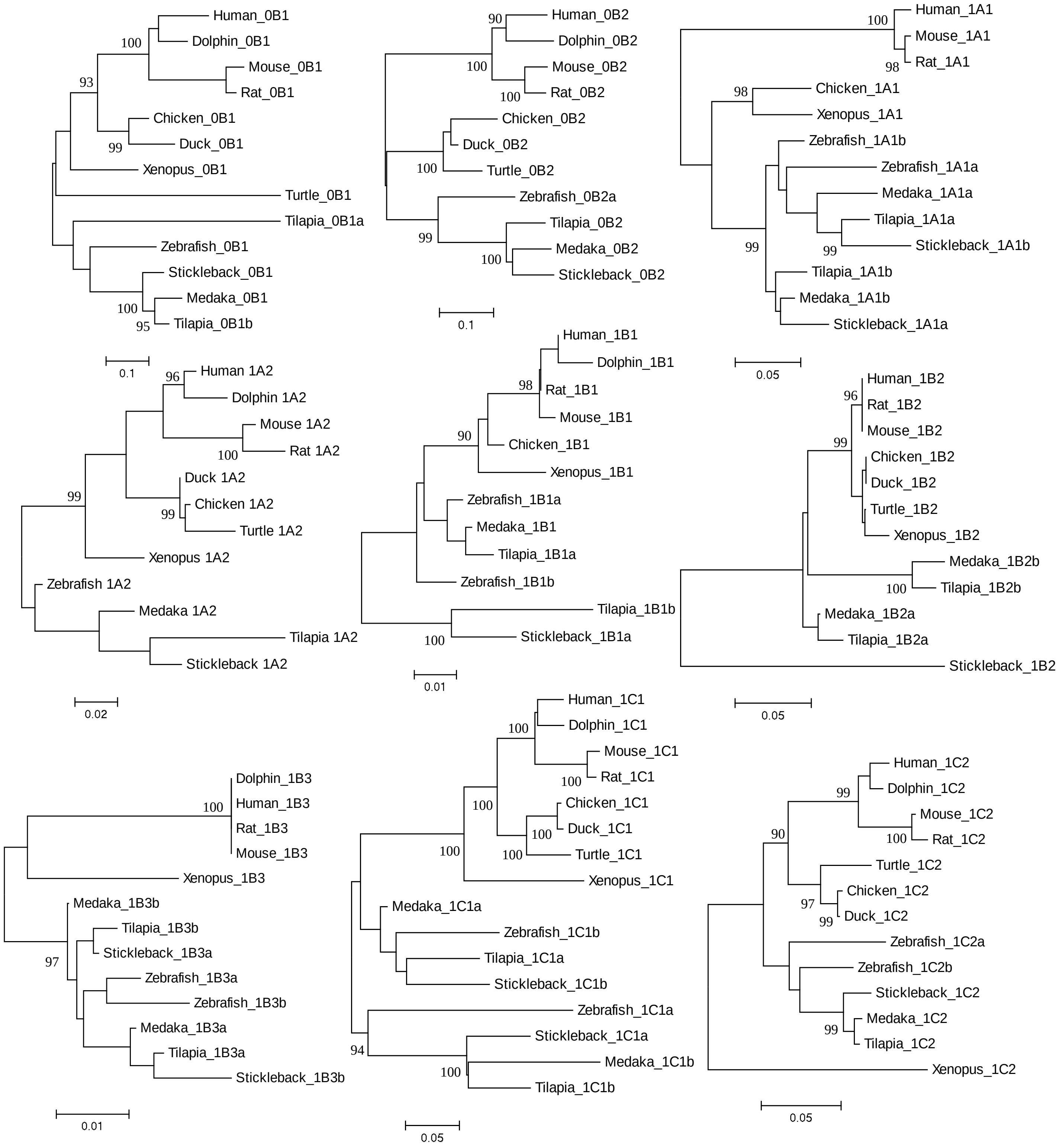

27

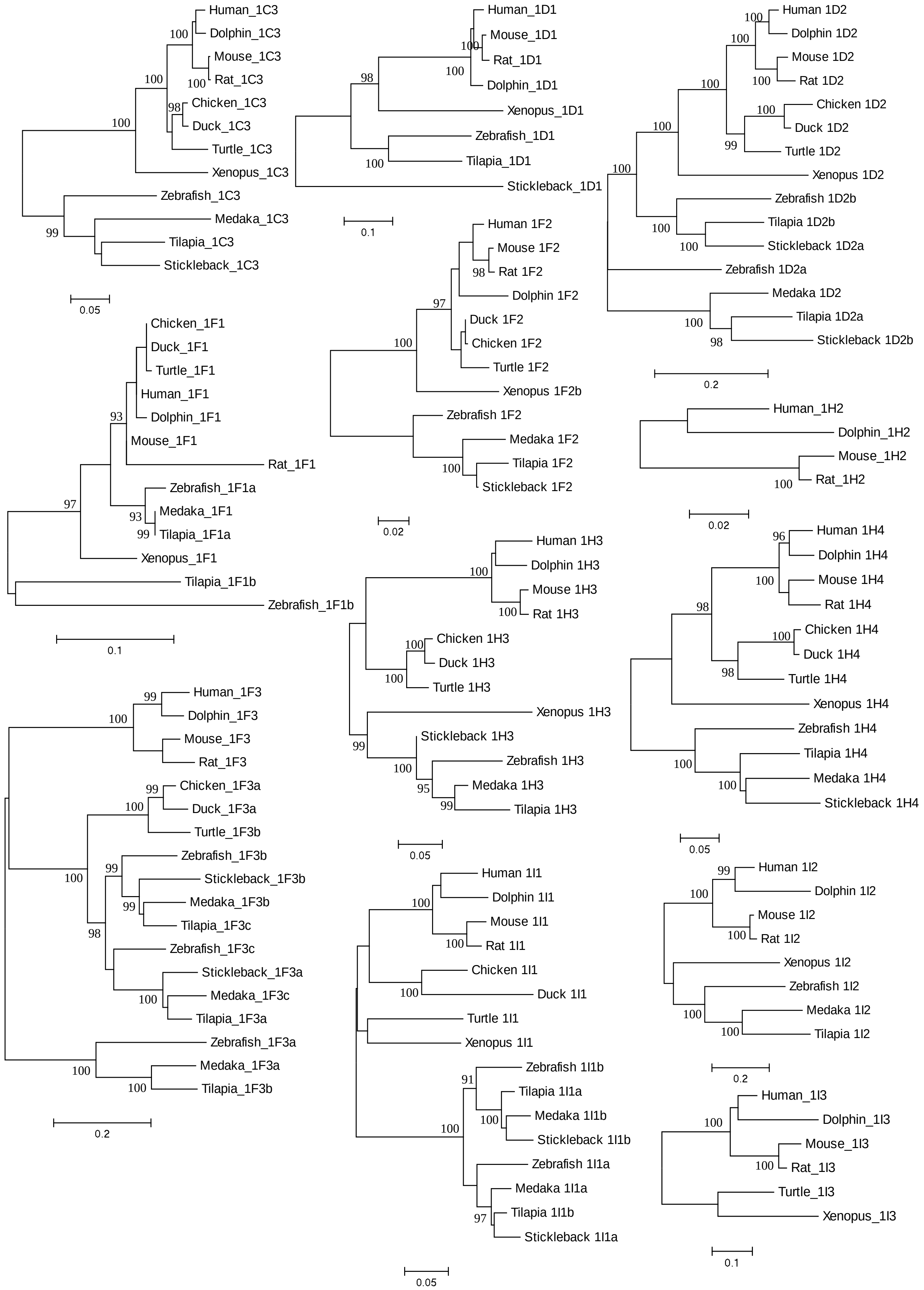

Human_1C3
Dolphin_1C3
Mouse_1C3
Rat_1C3
Chicken_1C3
Duck_1C3
Turtle_1C3
Xenopus_1C3
Zebrafish_1C3
Medaka_1C3
Tilapia_1C3
Stickleback_1C3
0.05
Human_1D1
Mouse_1D1
Rat_1D1
Dolphin_1D1
Xenopus_1D1
Zebrafish_1D1
Tilapia_1D1
Stickleback_1D1
0.1
Human 1D2
Dolphin 1D2
Mouse 1D2
Rat 1D2
Chicken 1D2
Duck 1D2
Turtle 1D2
Xenopus 1D2
Zebrafish 1D2b
Tilapia 1D2b
Stickleback 1D2a
Zebrafish 1D2a
Medaka 1D2
Tilapia 1D2a
Stickleback 1D2b
0.2
Chicken_1F1
Duck_1F1
Turtle_1F1
Human_1F1
Dolphin_1F1
Mouse_1F1
Rat_1F1
Zebrafish_1F1a
Medaka_1F1
Tilapia_1F1a
Xenopus_1F1
Tilapia_1F1b
Zebrafish_1F1b
0.1
Human 1F2
Mouse 1F2
Rat 1F2
Dolphin 1F2
Duck 1F2
Chicken 1F2
Turtle 1F2
Xenopus 1F2b
Zebrafish 1F2
Medaka 1F2
Tilapia 1F2
Stickleback 1F2
0.02
Human_1H2
Dolphin_1H2
Mouse_1H2
Rat_1H2
0.02
Human_1F3
Dolphin_1F3
Mouse_1F3
Rat_1F3
Chicken_1F3a
Duck_1F3a
Turtle_1F3b
Zebrafish_1F3b
Stickleback_1F3b
Medaka_1F3b
Tilapia_1F3c
Zebrafish_1F3c
Stickleback_1F3a
Medaka_1F3c
Tilapia_1F3a
Zebrafish_1F3a
Medaka_1F3a
Tilapia_1F3b
0.2
Human 1H3
Dolphin 1H3
Mouse 1H3
Rat 1H3
Chicken 1H3
Duck 1H3
Turtle 1H3
Xenopus 1H3
Stickleback 1H3
Zebrafish 1H3
Medaka 1H3
Tilapia 1H3
0.05
Human 1H4
Dolphin 1H4
Mouse 1H4
Rat 1H4
Chicken 1H4
Duck 1H4
Turtle 1H4
Xenopus 1H4
Zebrafish 1H4
Tilapia 1H4
Medaka 1H4
Stickleback 1H4
0.05
Human 1I1
Dolphin 1I1
Mouse 1I1
Rat 1I1
Chicken 1I1
Duck 1I1
Turtle 1I1
Xenopus 1I1
Zebrafish 1I1b
Tilapia 1I1a
Medaka 1I1b
Stickleback 1I1b
Zebrafish 1I1a
Medaka 1I1a
Tilapia 1I1b
Stickleback 1I1a
0.05
Human 1I2
Dolphin 1I2
Mouse 1I2
Rat 1I2
Xenopus 1I2
Zebrafish 1I2
Medaka 1I2
Tilapia 1I2
0.2
Human_1I3
Dolphin_1I3
Mouse_1I3
Rat_1I3
Turtle_1I3
Xenopus_1I3
0.1

28

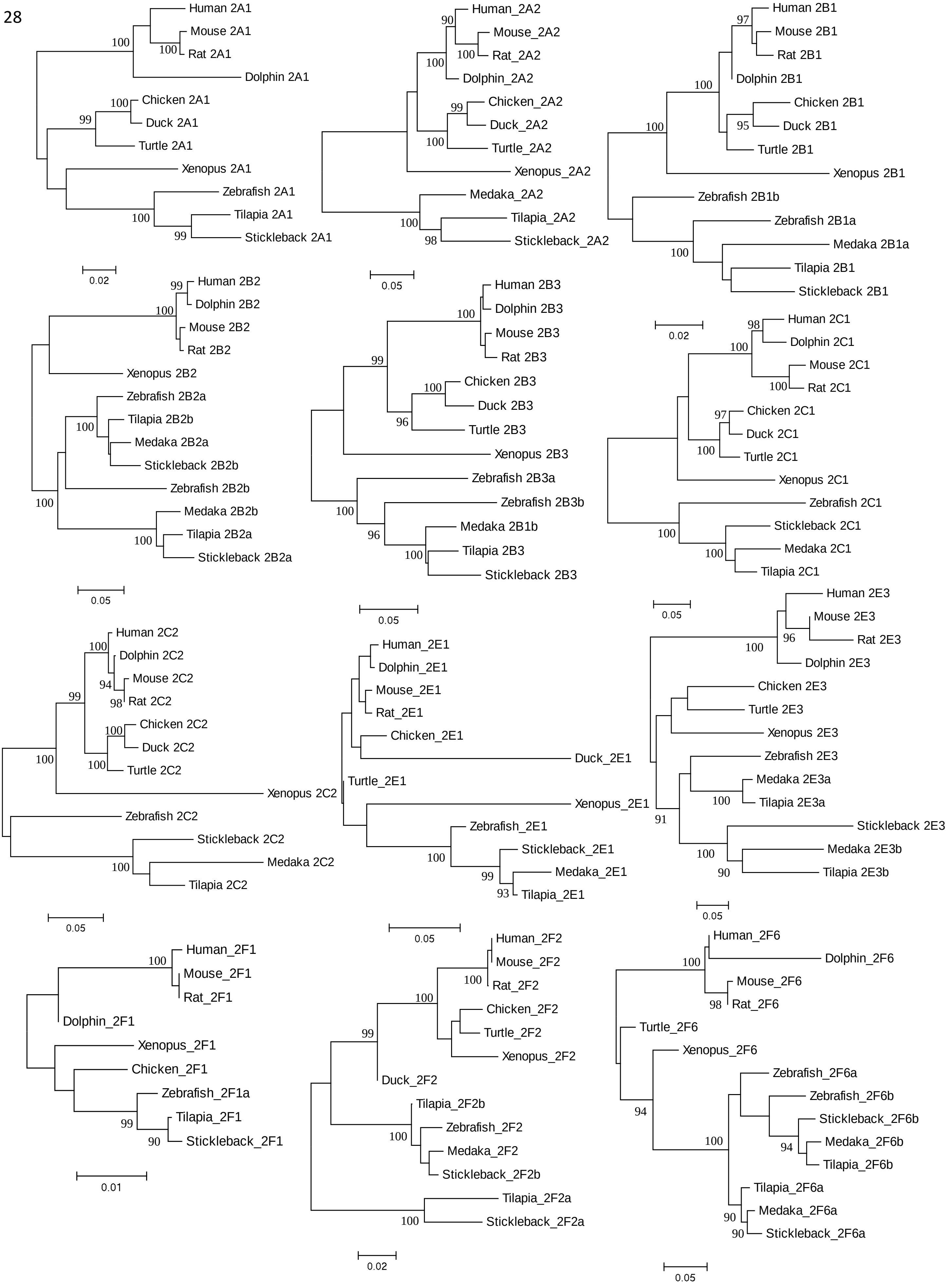
Human 2A1
Mouse 2A1
Rat 2A1
Dolphin 2A1
Chicken 2A1
Duck 2A1
Turtle 2A1
Xenopus 2A1
Zebrafish 2A1
Tilapia 2A1
Stickleback 2A1
0.02
Human_2A2
Mouse_2A2
Rat_2A2
Dolphin_2A2
Chicken_2A2
Duck_2A2
Turtle_2A2
Xenopus_2A2
Medaka_2A2
Tilapia_2A2
Stickleback_2A2
0.05
Human 2B1
Mouse 2B1
Rat 2B1
Dolphin 2B1
Chicken 2B1
Duck 2B1
Turtle 2B1
Xenopus 2B1
Zebrafish 2B1b
Zebrafish 2B1a
Medaka 2B1a
Tilapia 2B1
Stickleback 2B1
0.02
Human 2B2
Dolphin 2B2
Mouse 2B2
Rat 2B2
Xenopus 2B2
Zebrafish 2B2a
Tilapia 2B2b
Medaka 2B2a
Stickleback 2B2b
Zebrafish 2B2b
Medaka 2B2b
Tilapia 2B2a
Stickleback 2B2a
0.05
Human 2B3
Dolphin 2B3
Mouse 2B3
Rat 2B3
Chicken 2B3
Duck 2B3
Turtle 2B3
Xenopus 2B3
Zebrafish 2B3a
Zebrafish 2B3b
Medaka 2B1b
Tilapia 2B3
Stickleback 2B3
0.05
Human 2C1
Dolphin 2C1
Mouse 2C1
Rat 2C1
Chicken 2C1
Duck 2C1
Turtle 2C1
Xenopus 2C1
Zebrafish 2C1
Stickleback 2C1
Medaka 2C1
Tilapia 2C1
0.05
Human 2C2
Dolphin 2C2
Mouse 2C2
Rat 2C2
Chicken 2C2
Duck 2C2
Turtle 2C2
Xenopus 2C2
Zebrafish 2C2
Stickleback 2C2
Medaka 2C2
Tilapia 2C2
0.05
Human_2E1
Dolphin_2E1
Mouse_2E1
Rat_2E1
Chicken_2E1
Duck_2E1
Turtle_2E1
Xenopus_2E1
Zebrafish_2E1
Stickleback_2E1
Medaka_2E1
Tilapia_2E1
0.05
Human 2E3
Mouse 2E3
Rat 2E3
Dolphin 2E3
Chicken 2E3
Turtle 2E3
Xenopus 2E3
Zebrafish 2E3
Medaka 2E3a
Tilapia 2E3a
Stickleback 2E3
Medaka 2E3b
Tilapia 2E3b
0.05
Human_2F1
Mouse_2F1
Rat_2F1
Dolphin_2F1
Xenopus_2F1
Chicken_2F1
Zebrafish_2F1a
Tilapia_2F1
Stickleback_2F1
0.01
Human_2F2
Mouse_2F2
Rat_2F2
Chicken_2F2
Turtle_2F2
Xenopus_2F2
Duck_2F2
Tilapia_2F2b
Zebrafish_2F2
Medaka_2F2
Stickleback_2F2b
Tilapia_2F2a
Stickleback_2F2a
0.02
Human_2F6
Dolphin_2F6
Mouse_2F6
Rat_2F6
Turtle_2F6
Xenopus_2F6
Zebrafish_2F6a
Zebrafish_2F6b
Stickleback_2F6b
Medaka_2F6b
Tilapia_2F6b
Tilapia_2F6a
Medaka_2F6a
Stickleback_2F6a
0.05

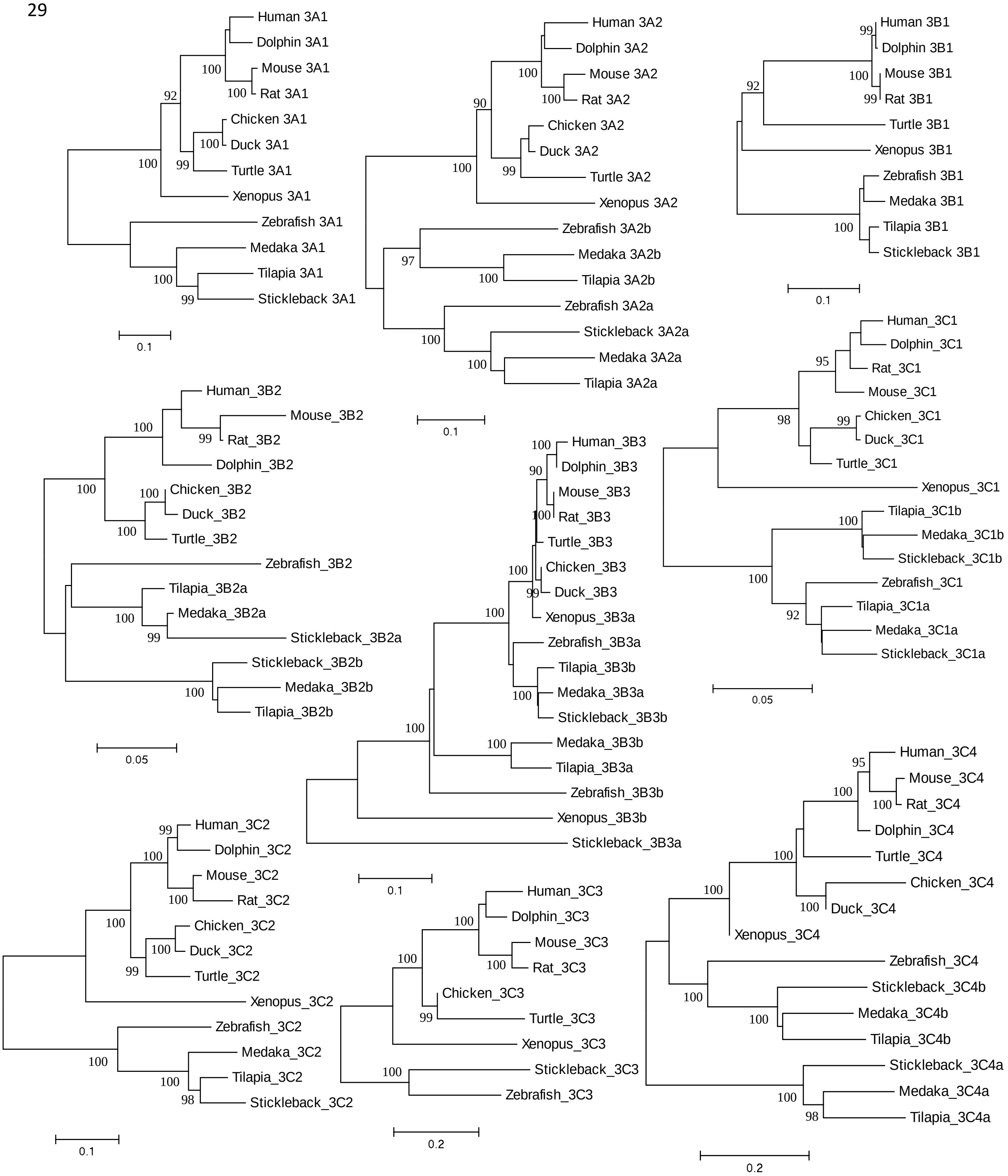
Human 3A1
Dolphin 3A1
Mouse 3A1
Rat 3A1
Chicken 3A1
Duck 3A1
Turtle 3A1
Xenopus 3A1
Zebrafish 3A1
Medaka 3A1
Tilapia 3A1
Stickleback 3A1
0.1
Human 3A2
Dolphin 3A2
Mouse 3A2
Rat 3A2
Chicken 3A2
Duck 3A2
Turtle 3A2
Xenopus 3A2
Zebrafish 3A2b
Medaka 3A2b
Tilapia 3A2b
Zebrafish 3A2a
Stickleback 3A2a
Medaka 3A2a
Tilapia 3A2a
0.1
Human 3B1
Dolphin 3B1
Mouse 3B1
Rat 3B1
Turtle 3B1
Xenopus 3B1
Zebrafish 3B1
Medaka 3B1
Tilapia 3B1
Stickleback 3B1
0.1
Human_3C1
Dolphin_3C1
Rat_3C1
Mouse_3C1
Chicken_3C1
Duck_3C1
Turtle_3C1
Xenopus_3C1
Tilapia_3C1b
Medaka_3C1b
Stickleback_3C1b
Zebrafish_3C1
Tilapia_3C1a
Medaka_3C1a
Stickleback_3C1a
0.05
Human_3B2
Mouse_3B2
Rat_3B2
Dolphin_3B2
Chicken_3B2
Duck_3B2
Turtle_3B2
Zebrafish_3B2
Tilapia_3B2a
Medaka_3B2a
Stickleback_3B2a
Stickleback_3B2b
Medaka_3B2b
Tilapia_3B2b
0.05
Human_3B3
Dolphin_3B3
Mouse_3B3
Rat_3B3
Turtle_3B3
Chicken_3B3
Duck_3B3
Xenopus_3B3a
Zebrafish_3B3a
Tilapia_3B3b
Medaka_3B3a
Stickleback_3B3b
Medaka_3B3b
Tilapia_3B3a
Zebrafish_3B3b
Xenopus_3B3b
Stickleback_3B3a
0.1
Human_3C4
Mouse_3C4
Rat_3C4
Dolphin_3C4
Turtle_3C4
Chicken_3C4
Duck_3C4
Xenopus_3C4
Zebrafish_3C4
Stickleback_3C4b
Medaka_3C4b
Tilapia_3C4b
Stickleback_3C4a
Medaka_3C4a
Tilapia_3C4a
0.2
Human_3C2
Dolphin_3C2
Mouse_3C2
Rat_3C2
Chicken_3C2
Duck_3C2
Turtle_3C2
Xenopus_3C2
Zebrafish_3C2
Medaka_3C2
Tilapia_3C2
Stickleback_3C2
0.1
Human_3C3
Dolphin_3C3
Mouse_3C3
Rat_3C3
Chicken_3C3
Turtle_3C3
Xenopus_3C3
Stickleback_3C3
Zebrafish_3C3
0.2

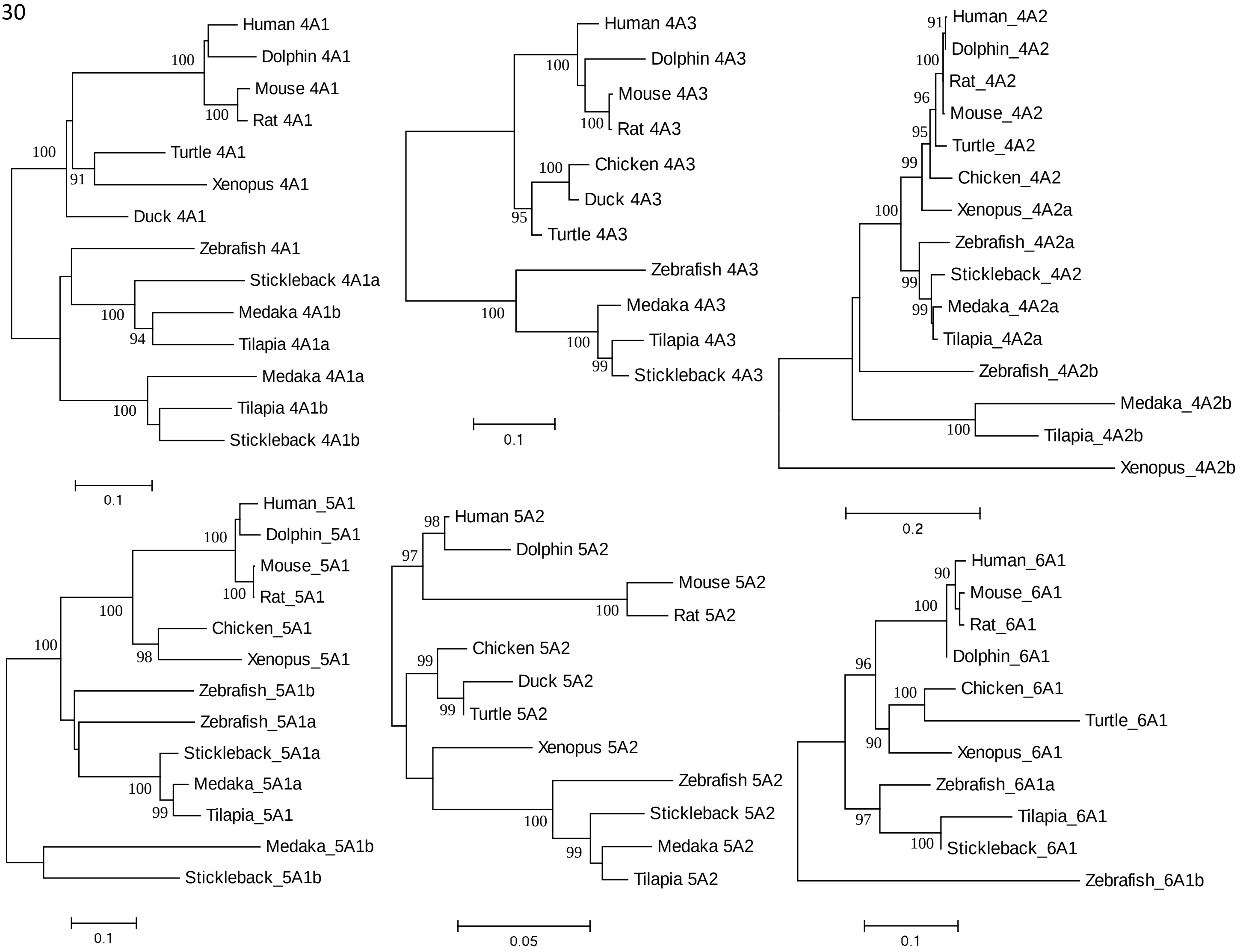
Human 4A1
Dolphin 4A1
Mouse 4A1
Rat 4A1
Turtle 4A1
Xenopus 4A1
Duck 4A1
Zebrafish 4A1
Stickleback 4A1a
Medaka 4A1b
Tilapia 4A1a
Medaka 4A1a
Tilapia 4A1b
Stickleback 4A1b
0.1
Human 4A3
Dolphin 4A3
Mouse 4A3
Rat 4A3
Chicken 4A3
Duck 4A3
Turtle 4A3
Zebrafish 4A3
Medaka 4A3
Tilapia 4A3
Stickleback 4A3
0.1
Human_4A2
Dolphin_4A2
Rat_4A2
Mouse_4A2
Turtle_4A2
Chicken_4A2
Xenopus_4A2a
Zebrafish_4A2a
Stickleback_4A2
Medaka_4A2a
Tilapia_4A2a
Zebrafish_4A2b
Medaka_4A2b
Tilapia_4A2b
Xenopus_4A2b
0.2
Human_5A1
Dolphin_5A1
Mouse_5A1
Rat_5A1
Chicken_5A1
Xenopus_5A1
Zebrafish_5A1b
Zebrafish_5A1a
Stickleback_5A1a
Medaka_5A1a
Tilapia_5A1
Medaka_5A1b
Stickleback_5A1b
0.1
Human 5A2
Dolphin 5A2
Mouse 5A2
Rat 5A2
Chicken 5A2
Duck 5A2
Turtle 5A2
Xenopus 5A2
Zebrafish 5A2
Stickleback 5A2
Medaka 5A2
Tilapia 5A2
0.05
Human_6A1
Mouse_6A1
Rat_6A1
Dolphin_6A1
Chicken_6A1
Turtle_6A1
Xenopus_6A1
Zebrafish_6A1a
Tilapia_6A1
Stickleback_6A1
Zebrafish_6A1b
0.1

Figure S2. Schematic diagram depicts the evolution of PXR and CAR in vertebrates.

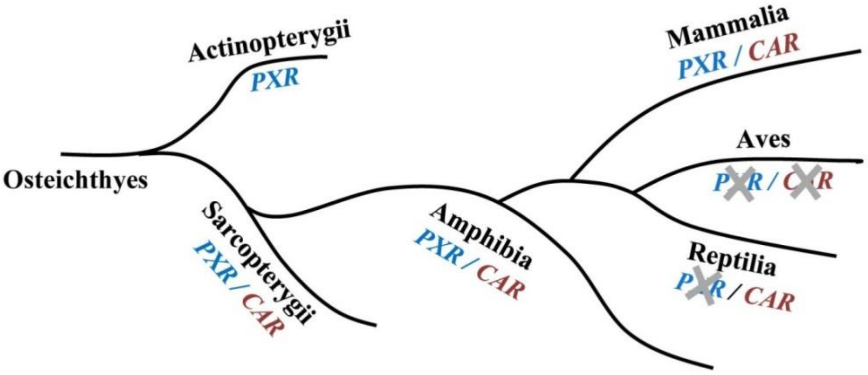

Table S1. Details for nuclear receptor sequence searches in 12 model vertebrates.

	BLASTn Hits	BLASTp Hits	Sum	After sortation	Verified by software	NR0B Subfamily	Final sets of NRs.
Human	33849	24967	58816	57	46	2	48
Mouse	23014	12540	35554	62	47	2	49
Rat	8312	8896	17208	70	47	2	49
Dolphin	2834	2752	5586	74	45	2	47
Chicken	3712	3761	7473	50	42	2	44
Duck	2381	4034	6415	48	40	2	42
Turtle	2922	3421	6343	48	46	2	48
Xenopus	2289	3850	6139	53	50	2	52
Zebrafish	9788	9230	19018	72	70	3	73
Medaka	3601	4090	7691	78	65	2	67
Tilapia	7586	6630	14216	83	71	3	74
Stickleback	571	268	839	64	64	2	66

Table S2. Sequence ID. for each nuclear receptor gene in Ensembl database.

	Human	Mouse	Rat	Dolphin	Chicken	Duck	Turtle	Xenopus	Zebrafish	Medaka	Tilapia	Stickleback
NR1A1	ENSG00000126351	ENSMUSG00000058756	ENSRNOG00000009066	ENSTTRG00000016893	ENSGALG00000000270	ENSAPLG00000016001	ENSPSIG00000012754	ENSXETG00000024399	ENSDARG00000000151	ENSORLG00000016941	ENSONIG00000018247	ENSGACG00000003766
									ENSDARG00000052654	ENSORLG00000012005	ENSONIG00000006456	ENSGACG00000006540
NR1A2	ENSG00000151090	ENSMUSG00000021779	ENSRNOG00000006649	ENSTTRG00000001859	ENSGALG00000011294	ENSAPLG00000006081	ENSPSIG00000008182	ENSXETG00000003871	ENSDARG00000021163	ENSORLG00000008122	ENSONIG00000010312	ENSGACG00000007996
NR1B1	ENSG00000131759	ENSMUSG00000037992	ENSRNOG00000009972	ENSTTRG00000016901	ENSGALG00000005629	ENSAPLG00000006377	ENSPSIG00000002372	ENSXETG00000024390	ENSDARG00000056783	ENSORLG00000004373	ENSONIG00000019915	ENSGACG00000012955
									ENSDARG00000034893		ENSONIG00000006314	ENSGACG00000005297
NR1B2	ENSG00000077092	ENSMUSG00000017491	ENSRNOG00000024061	ENSTTRG00000010874	ENSGALG00000011298	ENSAPLG00000006432	ENSPSIG00000007930	ENSXETG00000007272		ENSORLG00000008502	ENSONIG00000010320	ENSGACG00000007999
										ENSORLG00000016394	ENSONIG00000006493	
NR1B3	ENSG00000172819	ENSMUSG00000001288	ENSRNOG00000012499	ENSTTRG00000002778				ENSXETG00000012670	ENSDARG00000034117	ENSORLG00000015382	ENSONIG00000012223	ENSGACG00000009372
									ENSDARG00000054003	ENSORLG00000007861	ENSONIG00000019165	ENSGACG00000000612
NR1C1	ENSG00000186951	ENSMUSG00000022383	ENSRNOG00000021463	ENSTTRG00000004136	ENSGALG00000022985	ENSAPLG00000010641	ENSPSIG00000018221	ENSXETG00000023454	ENSDARG00000031777	ENSORLG00000002413	ENSONIG00000016715	ENSGACG00000018958
									ENSDARG00000054323	ENSORLG00000011091	ENSONIG00000008831	ENSGACG00000003703
NR1C2	ENSG00000112033	ENSMUSG00000002250	ENSRNOG00000000503	ENSTTRG00000009416	ENSGALG00000002588	ENSAPLG00000004751	ENSPSIG00000005889	ENSXETG00000015121	ENSDARG00000044525	ENSORLG00000006636	ENSONIG00000011871	ENSGACG00000008288
									ENSDARG00000009473			

NR1C3	ENSG00000132170	ENSMUSG00000000440	ENSRNOG00000008839	ENSTTRG00000016565	ENSGALG00000004974	ENSAPLG00000009031	ENSPSIG00000011100	ENSXETG00000017422	ENSDARG00000031848	ENSORLG00000004432	ENSONIG00000014331	ENSGACG00000001665
NR1D1	ENSG00000126368	ENSMUSG00000020889	ENSRNOG00000009329	ENSTTRG00000016894			ENSPSIG00000014806	ENSXETG00000024397	ENSDARG00000033160		ENSONIG00000009283	ENSGACG00000009356
NR1D2	ENSG00000174738	ENSMUSG00000021775	ENSRNOG00000046912	ENSTTRG00000010829	ENSGALG00000011291	ENSAPLG00000005753	ENSPSIG00000008488	ENSXETG00000003869	ENSDARG00000003820	ENSORLG00000016431	ENSONIG00000008699	ENSGACG00000012958
									ENSDARG00000009594		ENSONIG00000010308	ENSGACG00000007986
NR1D4									ENSDARG00000031161	ENSORLG00000007837	ENSONIG00000012213	ENSGACG00000000614
									ENSDARG00000059370	ENSORLG00000015399	ENSONIG00000019164	
NR1F1	ENSG00000069667	ENSMUSG00000032238	ENSRNOG00000027145	ENSTTRG00000007718	ENSGALG00000003759	ENSAPLG00000005866	ENSPSIG00000011314	ENSXETG00000021123	ENSDARG00000031768	ENSORLG00000007645	ENSONIG00000015289	
									ENSDARG00000001910		ENSONIG00000015603	
NR1F2	ENSG00000198963	ENSMUSG00000036192	ENSRNOG00000013413	ENSTTRG00000008387	ENSGALG00000015150	ENSAPLG00000007187	ENSPSIG00000005579	ENSXETG00000031251	ENSDARG00000033498	ENSORLG00000012441	ENSONIG00000010762	ENSGACG00000011556
								ENSXETG00000008148				
NR1F3	ENSG00000143365	ENSMUSG00000028150	ENSRNOG00000046831	ENSTTRG00000003151	ENSGALG00000025988	ENSAPLG00000013051	ENSPSIG00000008995	ENSXETG00000002131	ENSDARG00000087195	ENSORLG00000009486	ENSONIG00000004686	ENSGACG00000012280
					ENSGALG00000001035	ENSAPLG00000011493	ENSPSIG00000016262		ENSDARG00000057231	ENSORLG00000003765	ENSONIG00000010247	ENSGACG00000015341
									ENSDARG00000017780	ENSORLG00000014886	ENSONIG00000006222	
NR1H3	ENSG00000025434	ENSMUSG00000002108	ENSRNOG00000013172	ENSTTRG00000014149	ENSGALG00000008202	ENSAPLG00000010925	ENSPSIG00000010360	ENSXETG00000000307	ENSDARG00000043170	ENSORLG00000001286	ENSONIG00000005828	ENSGACG00000017167

NR1H2	ENSG00000131408	ENSMUSG00000060601	ENSRNOG00000019812	ENSTTRG00000002416								
NR1H5		ENSMUSG00000048938	ENSRNOG00000023073		ENSGALG00000002170	ENSAPLG00000008338	ENSPSIG00000003828	ENSXETG00000021443	ENSDARG00000031046		ENSONIG00000009252	ENSGACG00000004938
NR1H4	ENSG00000012504	ENSMUSG00000047638	ENSRNOG00000007197	ENSTTRG00000016373	ENSGALG00000011594	ENSAPLG00000013289	ENSPSIG00000005774	ENSXETG00000030372	ENSDARG00000057741	ENSORLG00000011270	ENSONIG00000014678	ENSGACG00000011745
NR1I1	ENSG00000111424	ENSMUSG00000022479	ENSRNOG00000008574	ENSTTRG00000012578	ENSGALG00000026166	ENSAPLG00000005087	ENSPSIG00000018108	ENSXETG00000010658	ENSDARG00000043059	ENSORLG00000001063	ENSONIG00000009200	ENSGACG00000004763
									ENSDARG00000070721	ENSORLG00000016402	ENSONIG00000019378	ENSGACG00000007975
NR1I2	ENSG00000144852	ENSMUSG00000022809	ENSRNOG00000002906	ENSTTRG00000016650				ENSXETG00000018029	ENSDARG00000029766	ENSORLG00000017953	ENSONIG00000014385	
NR1I3	ENSG00000143257	ENSMUSG00000005677	ENSRNOG00000003260	ENSTTRG00000009227	ENSGALG00000028624		ENSPSIG00000004437	ENSXETG00000031759				
NR2A1	ENSG00000101076	ENSMUSG00000017950	ENSRNOG00000008895	ENSTTRG00000013004	ENSGALG00000004285	ENSAPLG00000008950	ENSPSIG00000012689	ENSXETG00000001775	ENSDARG00000021494	ENSORLG00000016380	ENSONIG00000016515	ENSGACG00000011485
NR2A3					ENSGALG00000015670	ENSAPLG00000011331	ENSPSIG00000017650	ENSXETG00000016389	ENSDARG00000012764		ENSONIG00000005911	
NR2A2	ENSG00000164749	ENSMUSG00000017688	ENSRNOG00000008971	ENSTTRG00000003691	ENSGALG00000005708	ENSAPLG00000011794	ENSPSIG00000003756	ENSXETG00000017845	ENSDARG00000071565	ENSORLG00000006996	ENSONIG00000014490	ENSGACG00000002422
NR2B1	ENSG00000186350	ENSMUSG00000015846	ENSRNOG00000009446	ENSTTRG00000009492	ENSGALG00000002626	ENSAPLG00000013150	ENSPSIG00000011977	ENSXETG00000012733	ENSDARG00000057737	ENSORLG00000012155	ENSONIG00000013076	ENSGACG00000018189
									ENSDARG00000035127	ENSORLG00000016690		
NR2B2	ENSG00000204231	ENSMUSG00000039656	ENSRNOG00000000464	ENSTTRG00000004291				ENSXETG00000020416	ENSDARG00000078954	ENSORLG00000006476	ENSONIG00000020007	ENSGACG00000000096
									ENSDARG00000002006	ENSORLG00000007020	ENSONIG00000002873	ENSGACG00000007982

NR2B3	ENSG00000143171	ENSMUSG00000015843	ENSRNOG00000004537	ENSTTRG00000003653	ENSGALG00000003406	ENSAPLG00000004831	ENSPSIG00000004871	ENSXETG00000004750	ENSDARG00000005593		ENSONIG00000002143	ENSGACG00000011685
									ENSDARG00000004697			
NR2C1	ENSG00000120798	ENSMUSG00000005897	ENSRNOG00000006983	ENSTTRG00000016305	ENSGALG00000011327	ENSAPLG00000006253	ENSPSIG00000017190	ENSXETG00000023840	ENSDARG00000045527	ENSORLG00000004114	ENSONIG00000008566	ENSGACG00000010174
NR2C2	ENSG00000177463	ENSMUSG00000005893	ENSRNOG00000010536	ENSTTRG00000009876	ENSGALG00000008519	ENSAPLG00000007538	ENSPSIG00000008928	ENSXETG00000004817	ENSDARG00000042477	ENSORLG00000010877	ENSONIG00000017240	ENSGACG00000002941
NR2E1	ENSG00000112333	ENSMUSG00000019803	ENSRNOG00000050550	ENSTTRG00000008863	ENSGALG00000015305	ENSAPLG00000010675	ENSPSIG00000006035	ENSXETG00000014853	ENSDARG00000017107	ENSORLG00000013426	ENSONIG00000013281	ENSGACG00000008934
NR2E3	ENSG00000031544	ENSMUSG00000032292	ENSRNOG00000050690	ENSTTRG00000009410	ENSGALG00000002093		ENSPSIG00000017480	ENSXETG00000005219	ENSDARG00000045904	ENSORLG00000000011	ENSONIG00000007109	ENSGACG00000004739
										ENSORLG00000007175	ENSONIG00000015396	
NR2F1	ENSG00000175745	ENSMUSG00000069171	ENSRNOG00000014795	ENSTTRG00000001519	ENSGALG00000027907		ENSPSIG00000009818	ENSXETG00000011594	ENSDARG00000052695	ENSORLG00000010191	ENSONIG00000011840	ENSGACG00000010385
							ENSPSIG00000010198		ENSDARG00000017168			
NR2F2	ENSG00000185551	ENSMUSG00000030551	ENSRNOG00000010308		ENSGALG00000007000	ENSAPLG00000010629	ENSPSIG00000017164	ENSXETG00000022346	ENSDARG00000040926	ENSORLG00000008429	ENSONIG00000015133	ENSGACG00000013235
											ENSONIG00000003070	ENSGACG00000014846
NR2F5								ENSXETG00000011046	ENSDARG00000033172	ENSORLG00000016315	ENSONIG00000008594	ENSGACG00000013191
NR2F6	ENSG00000160113	ENSMUSG00000002393	ENSRNOG00000016892	ENSTTRG00000003132	ENSGALG00000027294	ENSAPLG00000003193	ENSPSIG00000013773	ENSXETG00000013531	ENSDARG00000003607	ENSORLG00000008749	ENSONIG00000010512	ENSGACG00000007766
									ENSDARG00000003165	ENSORLG00000008911	ENSONIG00000010104	ENSGACG00000015583

NR3A1	ENSG00000091831	ENSMUSG00000019768	ENSRNOG00000019358	ENSTTRG00000002996	ENSGALG00000012973	ENSAPLG00000004585	ENSPSIG00000004166	ENSXETG00000012364	ENSDARG00000004111	ENSORLG00000014514	ENSONIG00000013354	ENSGACG00000008711
NR3A2	ENSG00000140009	ENSMUSG00000021055	ENSRNOG00000005343	ENSTTRG00000000517	ENSGALG00000011801	ENSAPLG00000011895	ENSPSIG00000018210	ENSXETG00000007257	ENSDARG00000016454	ENSORLG00000017721	ENSONIG00000005633	ENSGACG00000007514
									ENSDARG00000034181	ENSORLG00000018012	ENSONIG00000001710	ENSGACG00000000213
NR3B1	ENSG00000173153	ENSMUSG00000024955	ENSRNOG00000021139	ENSTTRG00000010296			ENSPSIG00000016751	ENSXETG00000007211	ENSDARG00000069266	ENSORLG00000010624	ENSONIG00000001778	ENSGACG00000020287
NR3B2	ENSG00000119715	ENSMUSG00000021255	ENSRNOG00000010259	ENSTTRG00000001302	ENSGALG00000010365	ENSAPLG00000012470	ENSPSIG00000017916	ENSXETG00000013217	ENSDARG00000040151	ENSORLG00000016581	ENSONIG00000015282	ENSGACG00000010561
										ENSORLG00000009126	ENSONIG00000020192	ENSGACG00000007542
NR3B3	ENSG00000196482	ENSMUSG00000026610	ENSRNOG00000002593	ENSTTRG00000006004	ENSGALG00000009645	ENSAPLG00000005309	ENSPSIG00000005595	ENSXETG00000020932	ENSDARG00000004861	ENSORLG00000011528	ENSONIG00000000573	ENSGACG00000013426
								ENSXETG00000016948	ENSDARG00000011696	ENSORLG00000016819	ENSONIG00000017162	ENSGACG00000016275
NR3B4									ENSDARG00000015064		ENSONIG00000001134	ENSGACG00000004898
NR3C1	ENSG00000113580	ENSMUSG00000024431	ENSRNOG00000014096	ENSTTRG00000003260	ENSGALG00000007394	ENSAPLG00000007318	ENSPSIG00000015245	ENSXETG00000001879	ENSDARG00000025032	ENSORLG00000006022	ENSONIG00000017907	ENSGACG00000018209
										ENSORLG00000001565	ENSONIG00000008483	ENSGACG00000020725
NR3C2	ENSG00000151623	ENSMUSG00000031618	ENSRNOG00000034007	ENSTTRG00000014440	ENSGALG00000010035	ENSAPLG00000015146	ENSPSIG00000006383	ENSXETG00000026061	ENSDARG00000037025	ENSORLG00000007530	ENSONIG00000010029	ENSGACG00000017193
NR3C3	ENSG00000082175	ENSMUSG00000031870	ENSRNOG00000006831	ENSTTRG00000000030	ENSGALG00000017195	ENSAPLG00000003887	ENSPSIG00000013654	ENSXETG00000005482	ENSDARG00000035966	ENSORLG00000002651		ENSGACG00000012162
NR3C4	ENSG00000169083	ENSMUSG00000046532	ENSRNOG00000005639	ENSTTRG00000004230	ENSGALG00000004596	ENSAPLG00000006566	ENSPSIG00000010176	ENSXETG00000005089	ENSDARG00000067976	ENSORLG00000008220	ENSONIG00000012854	ENSGACG00000018525
										ENSORLG000	ENSONIG000	ENSGACG000

										00009520	00017538	00020332
NR4A1	ENSG00000123358	ENSMUSG00000023034	ENSRNOG00000007607	ENSTTRG00000002817		ENSAPLG00000014123	ENSPSIG00000018018	ENSXETG00000000579	ENSDARG00000000796	ENSORLG00000015557	ENSONIG00000016717	ENSGACG00000010788
										ENSORLG00000015279	ENSONIG00000019260	ENSGACG00000000045
NR4A2	ENSG00000153234	ENSMUSG00000026826	ENSRNOG00000005600	ENSTTRG00000005740	ENSGALG00000012538	ENSAPLG00000012071	ENSPSIG00000008054	ENSXETG00000031753	ENSDARG00000017007	ENSORLG00000016692	ENSONIG00000008976	ENSGACG00000005831
								ENSXETG00000024016	ENSDARG00000044532	ENSORLG00000000050	ENSONIG00000012131	
NR4A3	ENSG00000119508	ENSMUSG00000028341	ENSRNOG00000005964	ENSTTRG00000007458	ENSGALG00000013568	ENSAPLG00000011263	ENSPSIG00000012281		ENSDARG00000055854	ENSORLG00000008732	ENSONIG00000006026	ENSGACG00000009027
NR5A1	ENSG00000136931	ENSMUSG00000026751	ENSRNOG00000012682	ENSTTRG00000017390	ENSGALG00000001080	ENSAPLG00000004548	ENSPSIG00000006131	ENSXETG00000011456	ENSDARG00000017704	ENSORLG00000016486	ENSONIG00000020218	ENSGACG00000003539
									ENSDARG00000023362	ENSORLG00000013196		ENSGACG00000018317
NR5A2	ENSG00000116833	ENSMUSG00000026398	ENSRNOG00000000653	ENSTTRG00000003256	ENSGALG00000002182	ENSAPLG00000009302	ENSPSIG00000003632	ENSXETG00000000314	ENSDARG00000042556	ENSORLG00000006933	ENSONIG00000012517	ENSGACG00000008896
NR5A5									ENSDARG00000039116	ENSORLG00000006019	ENSONIG00000001686	ENSGACG00000009952
NR6A1	ENSG00000148200	ENSMUSG00000063972	ENSRNOG00000013232	ENSTTRG00000017391	ENSGALG00000001073	ENSAPLG00000004788	ENSPSIG00000006445	ENSXETG00000008578	ENSDARG00000018030	ENSORLG00000016492	ENSONIG00000020217	ENSGACG00000003560
									ENSDARG00000014480			
NR0B1	ENSG00000169297	ENSMUSG00000025056	ENSRNOG00000003765	ENSTTRG00000013272	ENSGALG00000016287	ENSAPLG00000003894	ENSPSIG00000009740	ENSXETG00000015374	ENSDARG00000056541	ENSORLG00000011824	ENSONIG00000012111	ENSGACG00000002817
											ENSONIG00000006662	
NR0B2	ENSG00000131910	ENSMUSG00000037583	ENSRNOG00000007229	ENSTTRG00000016680	ENSGALG00000000887	ENSAPLG00000010744	ENSPSIG00000017134	ENSXETG00000011771	ENSDARG00000044685	ENSORLG00000004442	ENSONIG00000006772	ENSGACG00000007198

Table S3. Genes with incomplete/without DBD/LBD regions in the Ensembl database. Genes marked in red means the full sequences were retrieved in NCBI/ EMBL/DDBJ databases.

	Gene and related Ensembl ID.
Human	—
Mouse	—
Rat	NR2E3 (ENSRNOG00000050690); NR3A1 (ENSRNOG00000019358); NR3C3 (ENSRNOG00000006831); NR5A2 (ENSRNOG00000000653)
Dolphin	NR1A1 (ENSTTRG00000016893); NR1B2 (ENSTTRG00000010874); NR1C1 (ENSTTRG00000004136); NR1F3 (ENSTTRG00000003151); NR1I2 (ENSTTRG00000016650); NR2A1 (ENSTTRG00000013004); NR2B1 (ENSTTRG00000009492); NR2B3 (ENSTTRG00000003653); NR2F6 (ENSTTRG00000003132); NR3A1 (ENSTTRG00000002996); NR3B2 (ENSTTRG00000001302); NR4A1 (ENSTTRG00000002817); NR4A3 (ENSTTRG00000007458)
Chicken	NR1B1 (ENSGALG00000005629); NR2F6 (ENSGALG00000027294);
Duck	NR1A1 (ENSAPLG00000016001); NR1B1(ENSAPLG00000006377); NR1F2 (ENSAPLG00000007187); NR1F3b (ENSAPLG00000011493); NR1H4 (ENSAPLG00000013289); NR1I1 (ENSAPLG00000005087); NR2F6 (ENSAPLG00000003193); NR3A1 (ENSAPLG00000004585); NR3C3 (ENSAPLG00000003887); NR4A1 (ENSAPLG00000014123); NR4A2 (ENSAPLG00000012071); NR5A1 (ENSAPLG00000004548); NR6A1 (ENSAPLG00000004788); NR0B1 (ENSAPLG00000003894)
Turtle	NR1A1 (ENSPSIG00000012754); NR1B1 (ENSPSIG00000002372); NR1D1 (ENSPSIG00000014806); NR2A2 (ENSPSIG00000003756); NR2E3 (ENSPSIG00000017480); NR2F1b (ENSPSIG00000010198); NR3B1 (ENSPSIG00000016751); NR4A2 (ENSPSIG00000008054); NR5A1 (ENSPSIG00000006131); NR6A1 (ENSPSIG00000006445)
Xenopus	NR1C2 (ENSXETG00000015121); NR1C3 (ENSXETG00000017422); NR1F3 (ENSXETG00000002131); NR2A1 (ENSXETG00000001775); NR4A2b (ENSXETG00000024016)
Zebrafish	NR1C3 (ENSDARG00000031848); NR1I1 (ENSDARG00000043059)
Medaka	NR1B1 (ENSORLG00000004373); NR2A1 (ENSORLG00000016380); NR2F1 (ENSORLG00000010191); NR3C3 (ENSORLG00000002651); NR6A1 (ENSORLG00000016492)
Tilapia	—
Stickleback	—

Table S4. The vertebrate species used for NR1I1 (VDR) gene phylogenetic analysis.

Common name	Scientific name	Common name	Scientific name
Human	*Homo sapiens*	Flycatcher	*Ficedula albicollis*
Gibbon	*Nomascus leucogenys*	Zebra Finch	*Taeniopygia guttata*
Gorilla	*Gorilla gorilla gorilla*	Duck	*Anas platyrhynchos*
Macaque	*Macaca mulatta*	Chicken	*Gallus gallus*
Marmoset	*Callithrix jacchus*	Turkey	*Meleagris gallopavo*
Bushbaby	*Otolemur garnettii*		
Cat	*Felis catus*	Anole lizard	*Anolis carolinensis*
Dog	*Canis lupus familiaris*	Chinese softshell turtle	*Pelodiscus sinensis*
Ferret	*Mustela putorius furo*		
Hedgehog	*Erinaceus europaeus*	Xenopus	*Xenopus tropicalis*
Rabbit	*Oryctolagus cuniculus*		
Dolphin	*Tursiops truncatus*	Coelacanth	*Latimeria chalumnae*
Pig	*Sus scrofa*	Tilapia	*Oreochromis niloticus*
Opossum	*Monodelphis domestica*	Zebrafish	*Danio rerio*
Cow	*Bos taurus*	Tetraodon	*Tetraodon nigroviridis*
Sheep	*Ovis aries*	Medaka	*Oryzias latipes*
Mouse	*Mus musculus*	Platyfish	*Xiphophorus maculatus*
Rat	*Rattus norvegicus*	Stickleback	*Gasterosteus aculeatus*
Guinea Pig	*Cavia porcellus*		
Squirrel	*Ictidomys tridecemlineatus*		
